# High-sensitivity multispeckle diffuse correlation spectroscopy

**DOI:** 10.1117/1.NPh.7.3.035010

**Published:** 2020-09-26

**Authors:** Edbert J. Sie, Hui Chen, E-Fann Saung, Ryan Catoen, Tobias Tiecke, Mark A. Chevillet, Francesco Marsili

**Affiliations:** Facebook Reality Labs Research, Menlo Park, California, United States

**Keywords:** diffuse correlation spectroscopy, near-infrared spectroscopy, single-photon avalanche photodiode camera, noninvasive

## Abstract

**Significance:** Cerebral blood flow is an important biomarker of brain health and function as it regulates the delivery of oxygen and substrates to tissue and the removal of metabolic waste products. Moreover, blood flow changes in specific areas of the brain are correlated with neuronal activity in those areas. Diffuse correlation spectroscopy (DCS) is a promising noninvasive optical technique for monitoring cerebral blood flow and for measuring cortex functional activation tasks. However, the current state-of-the-art DCS adoption is hindered by a trade-off between sensitivity to the cortex and signal-to-noise ratio (SNR).

**Aim:** We aim to develop a scalable method that increases the sensitivity of DCS instruments.

**Approach:** We report on a multispeckle DCS (mDCS) approach that is based on a 1024-pixel single-photon avalanche diode (SPAD) camera. Our approach is scalable to >100,000 independent speckle measurements since large-pixel-count SPAD cameras are becoming available, owing to the investments in LiDAR technology for automotive and augmented reality applications.

**Results:** We demonstrated a 32-fold increase in SNR with respect to traditional single-speckle DCS.

**Conclusion:** A mDCS system that is based on a SPAD camera serves as a scalable method toward high-sensitivity DCS measurements, thus enabling both high sensitivity to the cortex and high SNR.

## 1 Introduction

Measuring cerebral blood flow noninvasively and with high sensitivity is critical for clinical applications such as measuring the oxygen metabolic rate^1^^,^^2^ and monitoring intracranial pressure.^3^^,^^4^ Furthermore, although neuroscience applications such as functional activation mapping^5^^,^^6^ and noninvasive brain–computer interface^7^^,^^8^ have been pursued primarily using functional magnetic resonance imaging and near-infrared spectroscopy (fNIRS), such applications could in principle benefit from functional cerebral blood flow measurements.^9^^–^^11^ Diffuse correlation spectroscopy (DCS)^12^ is a promising noninvasive optical technique for monitoring cerebral blood flow^13^^,^^14^ and for measuring cortex functional activation during finger tapping^9^ and visual stimulation^10^^,^^11^ tasks. DCS measures deep-tissue dynamics by coupling coherent light into the subject and measuring the fluctuations in the speckle field created by the light diffusing out of the subject.^12^^,^^15^^,^^16^ Increasing the source–detector separation (ρ) of DCS optodes increases the proportion of detected photons that travel beneath the scalp and skull, deep into the brain cortex.^17^^–^^19^ However, the increase in sensitivity to deep tissue comes at the expense of a decrease in the signal-to-noise ratio (SNR) of the measured signal. Indeed, fewer diffuse photons reach the detector when the detector is farther away from the source due to absorption, scattering, and radial spread from the source. To increase the brain sensitivity of DCS, we developed multispeckle DCS (mDCS), a method that can extend ρ without sacrificing SNR by performing thousands of independent speckle measurements in parallel.

**Fig. 1:**
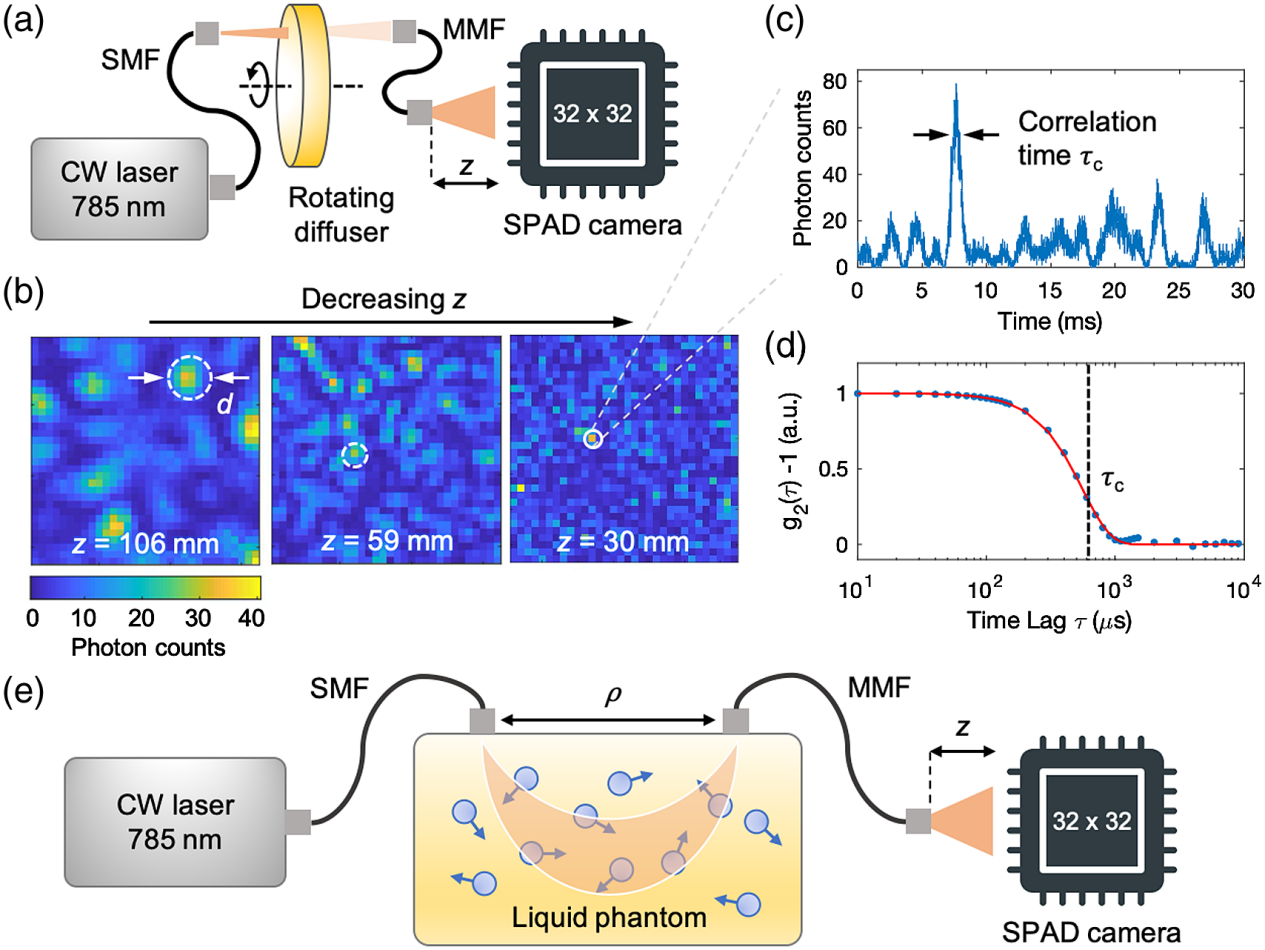
Experimental setup for multispeckle DCS. (a) Experimental setup consisting of a high-coherence-length (∼9  m) CW laser emitting at 785 nm wavelength, a diffuser plate, and a 32×32  pixel SPAD camera detector. The laser output was coupled to the phantom using an SMF. The diffused photons were collected by an MMF in a transmission geometry and coupled to the SPAD camera. We could change the fiber-SPAD distance (z) with a translating lens tube. The setup was used to calibrate the diameter of the speckles. The SPAD camera consisted of 1024 SPADs arranged in a 32×32 array, with a pixel pitch of 50×50  μm and a pixel active area of 6.95×6.95  μm. To measure the speckle turnover time trace shown in Fig. 1(c), we mounted the diffuser plate on a motorized rotation stage and rotated the plate at an angular speed of 15  deg/s. (b) Snapshots of speckle patterns recorded with the SPAD camera at varying z. These snapshots are recorded using the diffuser plate setup shown in Fig. 1(a). By adjusting the fiber-SPAD distance (z=106, 59, 30 mm), we can tune the average diameter of the speckles (d=8, 4, 2 pixels). The input laser power was tuned to avoid saturating the SPAD camera. Replacing the diffuser plate with a milk phantom did not change the diameter of the speckles, as long as we used the same fiber core diameter (D) and fiber-SPAD distance (z). The white circles denote the average diameter of the speckles that we estimated with 2D spatial autocorrelation (Appendix B). The frame exposure time was $T_{\text{bin}}=10\;\mu s$. (c) Detected photon counts versus time from a single pixel as shown in (b), recorded by rotating the diffuser plate around its axis. This time trace is the raw signal for calculating the intensity autocorrelation function $g_{2}(\tau )$. To resolve the dynamics of the speckles in this setup, we used $T_{\text{bin}}=10\;\mu s$, which is shorter than the correlation time of the speckles $\tau_{c}=557\;\mu s$. (d) Normalized intensity autocorrelation function $g_{2}(\tau )$ of the photon counts shown in (c). The $g_{2}$ curve was normalized by the value of $g_{2}$ at τ=10  μs. The correlation time constant $\tau_{c}$ (=557  μs) was estimated by fitting the data with the function $g_{2}(\tau )=1+\beta \;\exp(-\tau^{2}/\tau_{c}^{2})$ for a ballistic motion, where β is the coherence factor^15^^,^^20^ [see Appendix E for more details on the functional form of $g_{2}(\tau )$]. We normalized $g_{2}(\tau )-1$ and used $\tau_{c}$ as a fitting parameter. (e) Liquid phantom setup consisting of a source (SMF) and detector (MMF) optodes arranged in a reflection geometry. The source–detector separation (ρ) was 11 mm. This setup was used to measure the SNR gain and validate our mDCS model.

When we inject coherent light into a dynamic scattering medium such as those shown in Figs. 1(a) and 1(e), a dynamic speckle pattern emerges as shown in Fig. 1(b). DCS estimates the speed of the scatterers by measuring the correlation time constant of the speckles ($\tau_{c}$), which depends on the speed of the scatterers.^16^ We can estimate $\tau_{c}$ by measuring the intensity of a speckle versus time and by calculating the corresponding intensity autocorrelation function ($g_{2}$), as shown in Figs. 1(c) and 1(d).

Based on the correlation noise model,^21^^,^^22^ we can increase the SNR of the $g_{2}$ curve [defined as the ratio between mean and standard deviation of $g_{2}(\tau )$ calculated over several integration periods] by increasing the detected photon count rate ($N_{\mathrm{ph}}$) and the integration time (the time over which the intensity of the speckle field was recorded for each $g_{2}$ calculation, $T_{\text{int}}$). However, $T_{\text{int}}$ is limited by the time scales of the dynamics we need to measure (for example, $T_{\text{int}}$ should be ≲100  ms to measure pulsatility), and $N_{\mathrm{ph}}$ is limited by the skin and eye maximum permissible exposure (MPE) to laser radiation.^23^ Therefore, to increase SNR we need to consider parameters other than $T_{\text{int}}$ and $N_{\mathrm{ph}}$.

Traditional DCS systems employ single-mode fibers (SMFs) to couple one speckle onto a single-photon detector. There have been attempts to increase $N_{\mathrm{ph}}$ using a multimode fiber (MMF) to couple multiple speckles onto the same detector.^21^^,^^24^^,^^25^ Measuring multiple speckles on a single detector indeed increases $N_{\mathrm{ph}}$ linearly with the number of detected speckles, but at the same time it decreases the magnitude of $g_{2}-1$ (=β) by the same factor. Under typical experimental conditions for DCS, the increase in $N_{\mathrm{ph}}$ and the decrease in β compensate each other, so measuring multiple speckles with the same detector does not improve the SNR of $g_{2}$ (see Sec. 3.2 for more details on the SNR of DCS measurements). Therefore, we will henceforth refer to the above systems (one speckle/one detector and multiple speckles/one detector) as single-speckle DCS systems. mDCS is a new promising method to improve on the SNR of DCS through multiple independent measurements of the speckle field. The mDCS technique has been pursued in various ways using 28 stand-alone single-photon avalanche diodes (SPADs),^10^^,^^26^ 8 stand-alone SPADs,^27^ 4 pixels of a 5×5 SPAD array,^28^ and interferometric near-infrared spectroscopy detecting 20 speckles with a CMOS camera.^29^ While these prior approaches are important proofs of principle of mDCS, the largest improvement in SNR reported so far is an SNR gain (defined as the ratio between the SNR achieved with mDCS and with single-speckle DCS using detectors with the same performance) of 5.^26^ Here, we report on a method to perform 1024 independent DCS measurements using a kilopixel SPAD camera, which provides an SNR gain of 32. Our technique allows for DCS measurements with 36 times more speckles and approximately six times higher SNR gain with respect to the current state-of-the-art for mDCS. Our technique is ∼3 to 32 times more sensitive than single-speckle DCS using state-of-the-art high efficiency (∼70% at 780 nm) single photon detectors, depending on the count rate. With the ongoing development for megapixel SPAD technology,^30^^,^^31^ our approach offers a scalable implementation toward mDCS instruments with >10,000 times more speckles and >180 times higher SNR gain than the current state-of-the-art and >100 times higher SNR gain than single-speckle DCS.

## 2 Materials and Methods

### 2.1 Experimental Setup

Figures 1(a) and 1(e) show our experimental setup. We coupled a 785-nm wavelength CW laser to the scattering media with an SMF. We collected the light diffusing out of the scattering media with an MMF coupled to a SPAD camera (PF32, Photon-Force). The scattering media were a diffuser plate to calibrate the diameter of the speckles [Fig. 1(a)] and a liquid phantom to measure the SNR gain [Fig. 1(e)] (see Appendix A for details on the phantom setup).

To ensure that each pixel of the SPAD camera provided an independent measurement of the speckle field, we adjusted the average diameter of the speckles on the SPAD camera [d, see Fig. 1(b)] to be equal to or smaller than the size of the pixels of the SPAD camera (a=6.95  μm, see Appendix A for details on the SPAD camera). The average diameter of a speckle obeys the following relationship:^32^

$$d=\frac{\lambda z}{D},$$ (1)

where λ is the wavelength of the light (785 nm), z is the distance between the detector fiber end and the SPAD camera, and D is the fiber core diameter (910  μm). We calibrated our system by measuring d at different z with the setup shown in Fig. 1(a) (see Appendixes B and C for details on the speckle diameter calibration). Based on Eq. (1), the diameter d does not depend on the particular medium used, as long as D and z are fixed.

We measured the SNR gain of our mDCS system using a liquid phantom in a reflection geometry, as shown in Fig. 1(e), to better mimic the dynamics and optical properties of human tissue. The fiber-SPAD distance was z=8.1  mm, which corresponds to d=a. For the SNR gain measurements, the SPAD camera recorded the dynamics of the speckle field with a frame exposure time of $T_{\text{bin}}=4\;\mu s$ for up to ${2\times 10}^{6}$ frames (8 s).

### 2.2 Calculating the Intensity Autocorrelation Functions

We calculated the intensity autocorrelation function $g_{2}$ as a function of time lag τ for each pixel (i) of the camera:

$${g_{2}}^{i}(\tau )=\frac{\left\langle n(t)n(t+\tau )\right\rangle }{\left\langle n(t)\rangle \langle n(t+\tau )\right\rangle },$$ (2)

where n(t) is the number of detected photons in time bin t, τ is the time lag, and the square bracket ⟨…⟩ denotes the average over the integration time $T_{\text{int}}$.^33^^,^^34^ We calculated the ensemble average of the $g_{2}$ curves measured over M pixels as

$${{\overset{¯}{g}}_{2}\left(\tau \right)|}_{M}=\frac{1}{M}\overset{M}{\underset{i=1}{\sum }}{g_{2}}^{i}(\tau ).$$ (3)

## 3 Results and Discussion

### 3.1 SNR Gain using a SPAD Camera

**Fig. 2:**
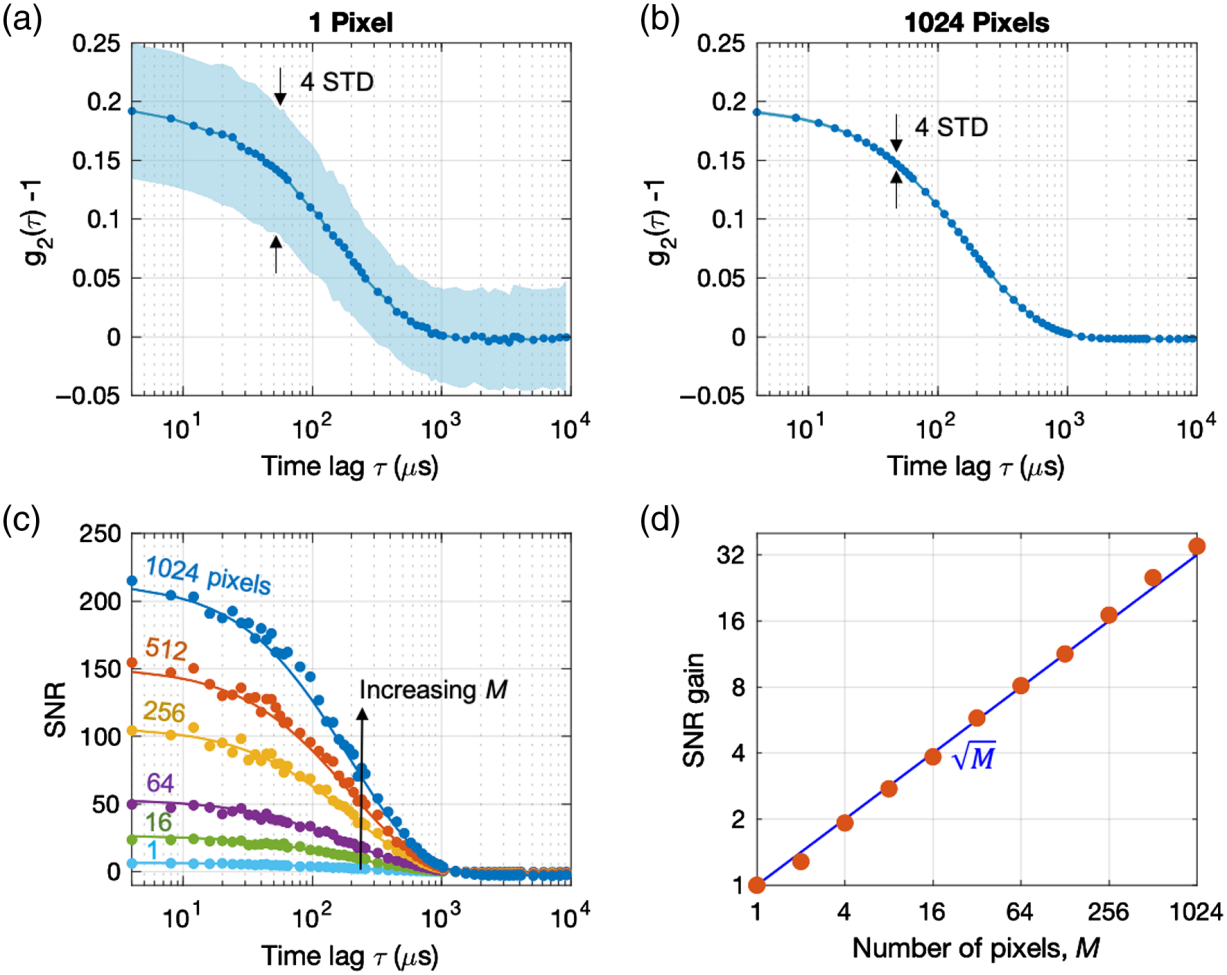
Achieving SNR gain of 32 using a 32×32  pixel SPAD camera. For this measurement, we used a CW laser at 785-nm wavelength coupled to the liquid phantom shown in Fig. 1(e). We used input power of 33 mW and ρ=11  mm to demonstrate the SNR gain using a SPAD camera. Under operating conditions using the MPE limit for skin (28.5 mW at 785 nm) and larger ρ, the SNR gain remains the same but the SNR magnitude depends on the measured count rate as we discussed in the next section. The fiber-SPAD distance was adjusted to z=8.1  mm to satisfy the condition d=a (see Appendix B for the estimation of d and Appendix C for the effect of d on the SNR^35^^,^^36^). (a) Time statistics of the pixel intensity autocorrelation function ${g_{2}}^{i}(\tau )$. The solid line corresponds to the mean value of ${g_{2}}^{i}(\tau )$ over 160 integration periods ($T_{\text{int}}=50\;\mathrm{ms}$) and the shade corresponds to ±2 standard deviations. The measured β=0.2 is lower than the value expected from the case of unpolarized light (β=0.5) because the speckle diameter matched the pixel active area (d=a)^35^ (see Appendix C). (b) Time statistics of the ensemble intensity autocorrelation function ${\overset{¯}{g}}_{2}(\tau )$. The solid line corresponds to the mean value of ${\overset{¯}{g}}_{2}(\tau )$ over 160 integration periods ($T_{\text{int}}=50\;\mathrm{ms}$). The standard deviation of ${\overset{¯}{g}}_{2}$ is 32 times smaller than that of ${g_{2}}^{i}$ and not as apparent in the plot. (c) SNR of ${\overset{¯}{g}}_{2}(\tau )$ for increasing size of the pixel ensemble M=1, 16, 64, 256, 512, and 1024. Averaging over a larger ensemble of pixels leads to higher SNR. The SNR measured over 160 integration periods (solid circles) is compared with the SNR calculated using the mDCS noise model with no fitting parameters (solid lines). All of the parameters of the noise model were obtained from experiments. (d) SNR gain of ${\overset{¯}{g}}_{2}(\tau =4\;\mu s)$ as a function of the size of the pixel ensemble M=1 to 1024. The SNR gain measured over 160 integration periods (solid circles) increases as the square root of the ensemble size (solid line), as predicted by our model.

Figure 2(a) shows a representative ${g_{2}}^{i}$ curve, and Fig. 2(b) shows the ensemble average measured over all of the pixels, ${{\overset{¯}{g}}_{2}|}_{M=1024}$. We measured $g_{2}$ with $T_{\text{int}}=50\;\mathrm{ms}$ and repeated the measurement for up to 8 s, for a total of 160 $g_{2}$ measurements per pixel. We calculated the time average $\left\{\mathrm{MEAN}[g_{2}(\tau )]\right\}$ and standard deviation $\left\{\mathrm{STD}[g_{2}(\tau )]\right\}$ of $g_{2}$ over all of the 160 integration periods. $\mathrm{STD}[{g_{2}}^{i}(\tau )]$ [Fig. 2(a)] was ∼32 times larger than the $\mathrm{STD}[{\overset{¯}{g}}_{2}(\tau )]$ [Fig. 2(b)] for the same $T_{\text{int}}$. Figure 2(c) shows the measured $\mathrm{SNR}[{\overset{¯}{g}}_{2}(\tau )]=\mathrm{MEAN}[{\overset{¯}{g}}_{2}(\tau )-1]/\mathrm{STD}[{\overset{¯}{g}}_{2}(\tau )]$ for increasing M (solid circles) and the SNR calculated using our mDCS noise model (to be discussed below) with no fitting parameters (solid lines). The SNR increased with increasing ensemble size, which is consistent with the prediction of our model. We estimated the SNR gain by calculating the ratio $\mathrm{SNR}[{\overset{¯}{g}}_{2}(\tau )]/\mathrm{SNR}[{g_{2}}^{i}(\tau )]$ for the first bin (τ=4  μs). Figure 2(d) shows that the measured SNR gain (solid circles) increased with the number of pixels as $\sqrt{M}$ (solid line) and reached a maximum value of 32 when we used all of the 1024 pixels of the camera, in agreement with our prediction.

### 3.2 Multispeckle DCS Noise Model

Zhou et al.^21^^,^^22^ reported on a noise model for single-speckle DCS that allows for estimating the SNR of $g_{2}$ from several experimental parameters that can be measured independently. To evaluate our findings quantitatively, we extended the noise model to mDCS by assuming that each speckle of the speckle field is an independent realization of the same ergodic random process (see below for details of the model). For ergodic random processes, ensemble and time statistics are the same, so observing one speckle for $T_{\text{int}}$ is equivalent to observing M speckles for $T_{\text{int}}/M$. Our model predicts that the SNR of the first bin (τ=4  μs) is proportional to $N_{\mathrm{ph}}\times \sqrt{T_{\mathrm{int}}}\times \sqrt{M}$ in the shot-noise limited regime, in which most *in vivo* DCS measurements operate (see below for details of the noise components of the speckle field).

Under the assumption of speckle ergodicity, we extended the single-speckle DCS noise model^21^^,^^22^ to mDCS by including the number of independent speckle field measurements (M) alongside the integration time ($T_{\text{int}}$) in the expression for $\mathrm{STD}[{\overset{¯}{g}}_{2}(\tau )]$:

$$\mathrm{STD}[{\overset{¯}{g}}_{2}(\tau )]=\sqrt{\frac{T_{\text{bin}}}{T_{\text{int}}M}}[{\left\langle n\right\rangle }^{-2}(1+\beta e^{-\tau /2\tau_{c}})+2{\left\langle n\right\rangle }^{-1}\beta (1+e^{-\tau /\tau_{c}})\;+{\beta^{2}\frac{(1+e^{-T_{\text{bin}}/\tau_{c}})(1+e^{-\tau /\tau_{c}})+2m(1-e^{-T_{\text{bin}}/\tau_{c}})e^{-\tau /\tau_{c}}}{\left(1-e^{-T_{\text{bin}}/\tau_{c}}\right)}]}^{1/2},$$ (4)

where $T_{\text{bin}}$ is the frame exposure time (which is equal to the correlator time bin interval), $T_{\text{int}}$ is the integration time, M is the number of independent speckle field measurements, β is the coherence factor, $\tau_{c}$ is the speckle correlation time, m is the delay time bin index such that $\tau ={mT}_{\text{bin}}$, and ⟨n⟩ ($=N_{\mathrm{ph}}\times T_{\text{bin}}$) is the number of detected photons within $T_{\text{bin}}$ per pixel. To derive the expression for $\mathrm{STD}[{\overset{¯}{g}}_{2}(\tau )]$ shown in Eq. (4), we simplified the intensity autocorrelation function with ${\overset{¯}{g}}_{2}(\tau )\approx 1+\beta \;\exp(-\tau /\tau_{c})$, which is a good approximation under the operating conditions of our liquid phantom measurements [see Appendix E for more details on the functional forms of $g_{2}(\tau )$].

**Fig. 3:**
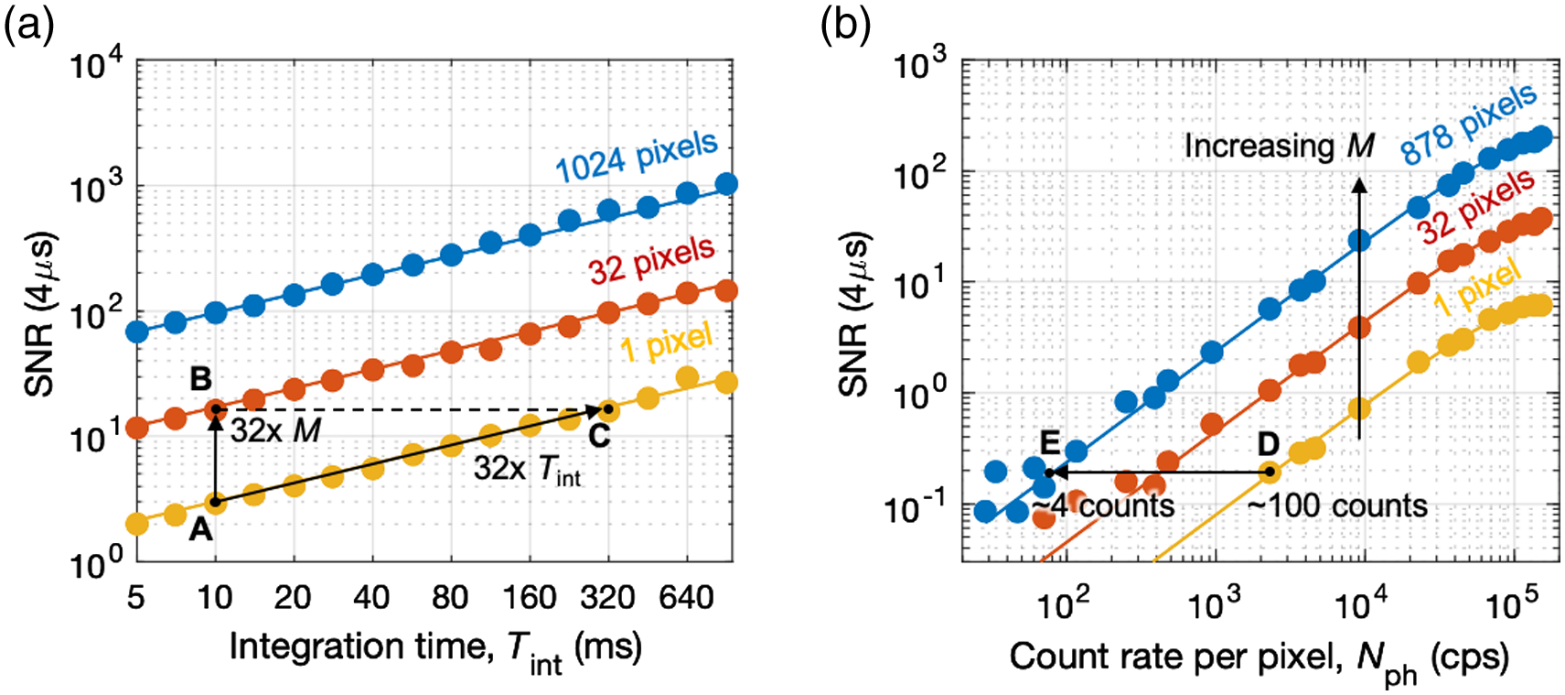
mDCS noise model. The fiber-SPAD distance was adjusted to z=8.1  mm to satisfy the condition d=a. The SNR is the ratio between the mean and the standard deviation of ${\overset{¯}{g}}_{2}$ measured over 160 integration periods. (a) SNR (τ=4  μs) of ${\overset{¯}{g}}_{2}$ for increasing integration times $T_{\text{int}}$ and fixed $N_{\mathrm{ph}}=1.5\times 10^{5}\;\mathrm{cps}$, measured (solid circles) and calculated (solid lines) using different pixel ensemble sizes M=1, 32, and 1024. The SNR gain achieved by increasing the ensemble size to M=32 (see arrow from points A to B) can also be achieved by increasing $T_{\text{int}}$ by 32 times (see arrow from points A to C). (b) SNR (τ=4  μs) of ${\overset{¯}{g}}_{2}$ for increasing detected photon count rates $N_{\mathrm{ph}}$ and fixed $T_{\text{int}}=50\;\mathrm{ms}$, measured (solid circles) and calculated (solid lines) using different pixel ensemble sizes M=1, 32, and 878. The SNR measured with only one pixel and ∼100 detected photons per $T_{\text{int}}$ (point D) is comparable to the SNR measured by averaging over 878 pixels and as low as ∼4 detected photons per pixel per $T_{\text{int}}$ (point E). Here, we used only up to M=878  pixels to avoid the DCR contribution from hot pixels (see Appendix D for details on DCR characterizations on the SPAD camera).

We write Eq. (4) in the following form:

$$\mathrm{STD}[{\overset{¯}{g}}_{2}(\tau )]=\frac{1}{\left\langle n\right\rangle }\sqrt{\frac{T_{\text{bin}}}{T_{\text{int}}M}}{\left[A(\tau )+B(\tau )\langle n\rangle +C(\tau ){\left\langle n\right\rangle }^{2}\right]}^{\left(1/2\right)},$$ (5)

where A, B, and C are functions of τ and other correlation parameters. In the limit of low photon count rates ($\langle n\rangle \ll \sqrt{A/C}$), A dominates, which leads to $\mathrm{SNR}[{\overset{¯}{g}}_{2}(\tau \to 0)]=\langle n\rangle \sqrt{\frac{T_{\mathrm{int}}M}{T_{\mathrm{bin}}}\frac{\beta^{2}}{\left(1+\beta \right)}}$. Therefore, for low count rates, the SNR is proportional to $N_{\mathrm{ph}}\times \sqrt{T_{\mathrm{int}}}\times \sqrt{M}$, and it is limited by the shot noise of the speckle field (fluctuation in the number of photons in a speckle). At higher photon count rates ($\langle n\rangle \gg \sqrt{A/C}$), C dominates, which leads to $\mathrm{SNR}[{\overset{¯}{g}}_{2}(\tau \to 0)]=\sqrt{T_{\mathrm{int}}M/(4\tau_{c})}$. Therefore, for high count rates, the SNR is limited by the speckle noise (fluctuation in the coherence time of a speckle) and independent of the $N_{\mathrm{ph}}$. The SNR transitions between the shot-noise and speckle-noise limited regimes at the threshold count rate, $N_{\mathrm{th}}=\frac{1}{T_{\text{bin}}}\sqrt{A/C}$, which is apparent from the gradual changes in the slope of the SNR versus $N_{\mathrm{ph}}$ curves as shown in Fig. 3(b).

The mDCS noise model is based on the assumption that the dominant source of noise is the intrinsic statistical temporal fluctuation of the speckle field, which is due to shot noise and speckle noise. Thus, the validity of the noise model is limited to measurements with negligible extrinsic noise sources, such as dark counts from the detector, after-pulsing, pixel cross-talk, and background photons. Depending on the relative magnitudes of extrinsic and intrinsic noises, increasing the number of speckles coupled to a pixel (d<a) may have different effects on the SNR of $g_{2}$. If shot noise is dominant, measuring multiple speckles on a single detector increases $N_{\mathrm{ph}}$ linearly with the number of detected speckles, but at the same time it decreases β by the same factor. Since these two effects approximately compensate each other, measuring multiple speckles with the same detector does not improve the SNR of $g_{2}$. If speckle noise is dominant, the number of speckles coupled to a pixel does not affect the SNR because the SNR does not depend on $N_{\mathrm{ph}}$ or β. Finally, if extrinsic noise is dominant, coupling multiple speckles to a pixel may increase the SNR by increasing $N_{\mathrm{ph}}$.

Since $\tau_{c}$ in brain tissue is in the range of tens of μs, which becomes shorter for larger source–detector distances ρ,^26^ most *in vivo* DCS measurements are likely to operate in the shot-noise limited regime. For instance, for $T_{\text{bin}}=1\;\mu s$, τ=1  μs, $\tau_{c}=10\;\mu s$, and β=0.5, the threshold count rate is $N_{\mathrm{th}}∼10^{5}\;\mathrm{cps}\;\mathrm{per}\;\text{pixel}$, which is over an order of magnitude larger than the count rates reported for DCS measurements on an adult human head at ρ=29  mm ($∼10^{4}\;\mathrm{cps}\;\mathrm{per}$ speckle).^26^

To support our ergodicity assumption and validate our model experimentally, we compared the SNR of ${\overset{¯}{g}}_{2}$ and ${g_{2}}^{i}$ at τ=4  μs for increasing $T_{\text{int}}$ or $N_{\mathrm{ph}}$. Figure 3(a) shows the SNR versus $T_{\text{int}}$ with different M=1, 32, and 1024. Increasing the ensemble size by 32 times from 1 to 32 at $T_{\text{int}}=10\;\mathrm{ms}$ (see arrow from point A to point B) resulted in the same SNR gain that would be achieved with one pixel and 32 times longer $T_{\text{int}}$ (see arrow from point A to point C), as predicted by our model. This result supports our ergodicity hypothesis that pixel ensemble averaging and time averaging result in the same SNR gain. Similarly, the SNR versus $N_{\mathrm{ph}}$ curves in Fig. 3(b) show that increasing the ensemble size by 878 times is equivalent to increasing the count rate of one pixel by ∼30 times. The solid lines in Fig. 3 are predictions based on our model with no fitting parameters.

The measurements shown in Fig. 3(b) also illustrate how our ensemble averaging technique is applicable to photon-starved applications, such as the detection of deep-tissue dynamics. Figure 3(b) shows that the SNR measured with only one pixel and ∼100 detected photons per $T_{\text{int}}$ (point D) is comparable to the SNR measured by averaging over 878 pixels and as low as ∼4 detected photons per pixel per $T_{\text{int}}$ (point E). mDCS reached the predicted SNR gain even in the case of an unprecedentedly small number of detected photons available to calculate $g_{2}$ for each pixel.

In summary, Figs. 2(c), 2(d), 3(a), and 3(b) show that our mDCS noise model (solid lines) could predict the experimental results (solid circles) well without any fitting parameters. In particular, we confirmed experimentally the mDCS noise model prediction that the SNR is proportional to $N_{\mathrm{ph}}\times \sqrt{T_{\mathrm{int}}}\times \sqrt{M}$ in the shot-noise limit. Our results confirm that we can increase the SNR beyond the limits imposed by the integration time $T_{\text{int}}$ and the photon count rate $N_{\mathrm{ph}}$ by increasing the ensemble size M.

## 4 Conclusions and Outlook

We have demonstrated a scalable method for mDCS measurements to enhance the SNR gain by a factor of 32 with respect to single-speckle DCS using a kilopixel SPAD camera. Our technique can be used to achieve higher sensitivity to the cortex than traditional DCS using a larger source–detector distance (ρ), while maintaining higher SNR. We have also extended the DCS noise model to the case of multiple independent speckle field measurements and validated our model experimentally. Our results indicate that our mDCS model works well even in extremely photon-starved conditions (down to four detected photons per pixel available to calculate $g_{2}$), thus enabling measurements at a large ρ.

Due to the recent investments in LiDAR technology for automotive and consumer electronics applications, high-performance large-pixel-count SPAD cameras with improved detection efficiency are rapidly becoming more available and less expensive.^30^^,^^37^ While the first megapixel SPAD array was recently reported,^31^ for mDCS to take full advantage of the extraordinary advances of SPAD camera technology, we envision the need for real-time data compression schemes implemented in the read-out FPGA of the SPAD camera or directly on chip.^30^ Finally, our mDCS technique can also be implemented in the time domain to enhance sensitivity to deep tissue.^38^^–^^41^

## 5 Appendix A: Experimental Setup

In our experiments, we used a high-coherence-length (∼9  m), 785-nm wavelength CW laser (Thorlabs, DBR785P) that was coupled to dynamic scattering media with an SMF (5-μm core diameter, 730-nm cutoff wavelength). The source power was adjusted by an attenuator to produce up to 33 mW at the output of the SMF. We collected the light diffusing out of the dynamic scattering media with an MMF (910-μm core diameter, 0.22 NA) coupled to a 32×32  pixel SPAD camera (Photon-Force, PF32). The SPAD camera consisted of 1024 SPADs arranged in a 32×32 array, with a pixel pitch of 50×50  μm and a pixel active area of 6.95×6.95  μm. Each pixel was surrounded by electronics necessary to bias and quench the SPAD, as well as to record photon detection events, with a detection efficiency of ∼8.2% at an excess bias of 1.7 V and wavelength of 785 nm. About ∼85% of the pixels had an average dark count rate (DCR) of ∼24  Hz. Each pixel was configured to record between 0 and 127 detection events per frame. We ran the measurements in the photon-counting mode using frame exposure times in the range of $4\;\mu s<T_{\text{bin}}<10\;\mu s$, with the corresponding frame rate between 250 and 100 kfps. As shown in Fig. 1, our scattering media were a diffuser plate to calibrate the diameter of the speckles [Fig. 1(a)] and a liquid phantom to measure the SNR gain [Fig. 1(e)].

The diffuser plate consisted of a 1-in.-diameter ground glass diffuser (Thorlabs, DG10-120). The source fiber (SMF), the diffuser phantom, and the detector fiber (MMF) were arranged in a transmission geometry with a 10-mm gap between the phantom and the detector optode. The other end of the detector fiber was coupled to the SPAD camera with a translating lens tube that allowed us to adjust the MMF-SPAD distance in the range of z=3.5 to 200 mm. The image of the speckles was recorded using the SPAD camera at $T_{\text{bin}}=10\;\mu s$. The diameter of the speckles was determined using two-dimensional (2D) spatial autocorrelation of the speckle pattern as described in Appendix B. To measure the speckle turnover time trace, we mounted the diffuser plate on a motorized rotation stage and rotated the plate at an angular speed of 15  deg/s.

The liquid phantom was a mixture of milk and water with a volume ratio of 1:8 in a $19\times 19\times 21\;{\mathrm{cm}}^{3}$ black Noryl plastic container at room temperature. The temperature of the room was 21±0.5°C. We did not actively stabilize the temperature of the milk phantom. We brought the connectors of the source (SMF) and detector (MMF) fibers into contact with the surface of the mixture. The source–detector distance was ρ=11  mm. The other end of the MMF optode was coupled to the SPAD camera with an MMF-SPAD distance of z=8.1  mm to achieve d=a. The frame exposure time was $T_{\text{bin}}=4\;\mu s$.

## 6 Appendix B: Calibrating the Diameter of the Speckles Using 2D Spatial Autocorrelation

**Fig. 4:**
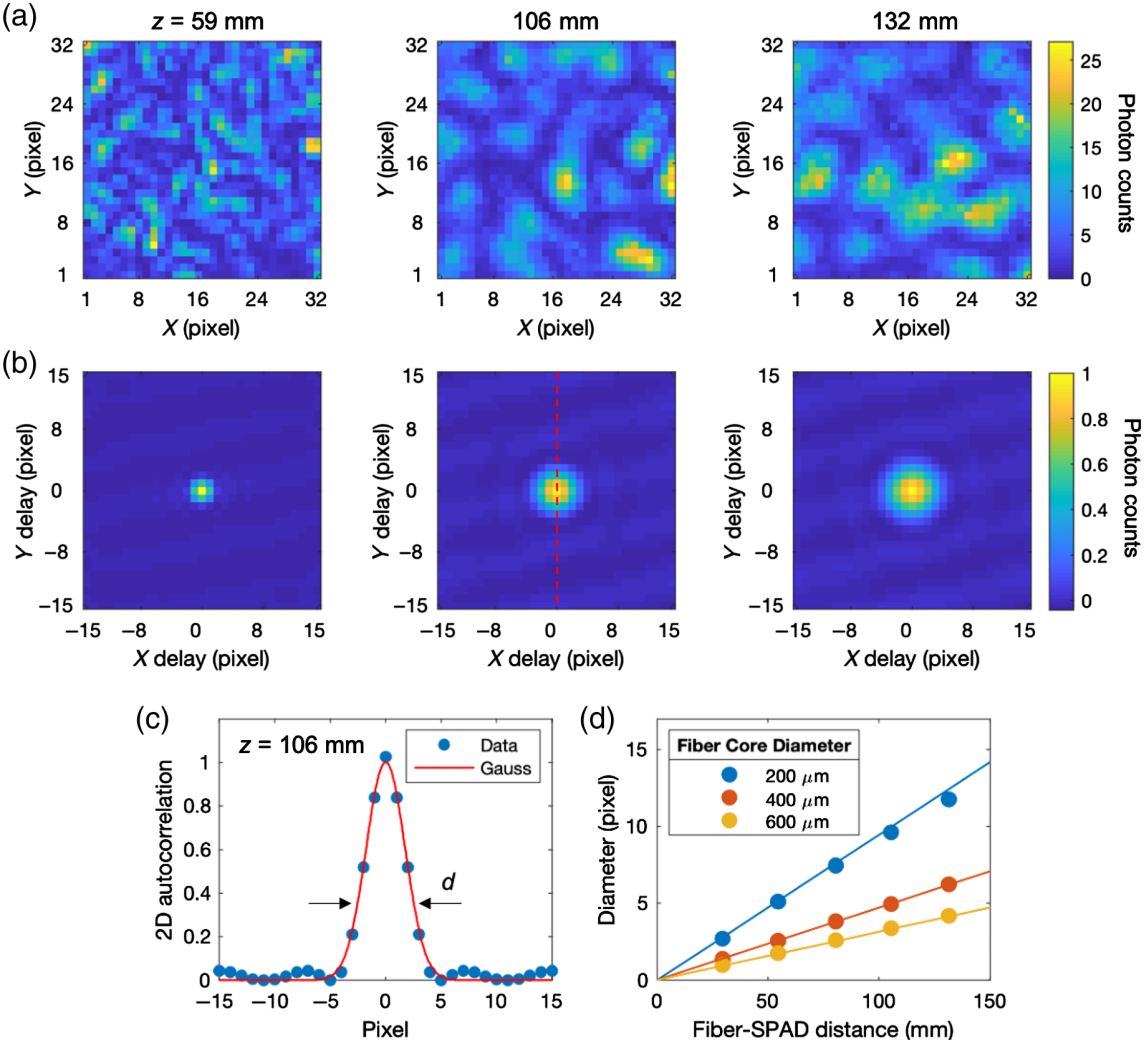
Measuring the diameter of the speckles using 2D spatial autocorrelation. (a) Snapshots of the speckle pattern at increasing fiber-SPAD distances z=59, 106, and 132 mm. These snapshots show that the average size of the speckles gets larger at increasing z. (b) 2D spatial autocorrelation images of the speckle pattern from (a). These images provide more quantitative information about the diameter of the speckles at respective z. (c) A linecut of the 2D spatial autocorrelation images from the z=106  mm in (b). We estimated the measured average diameter of the speckle (solid circles) through Gaussian fitting (solid curve). (d) Measured diameter of the speckles (solid circles) at varying detector MMF fiber core diameters. The straight lines are comparison based on the d=λz/D calculation.

In this experiment, we used a diffuser plate in transmission geometry with a 785-nm CW laser source, an SM source fiber optode (4.4-μm core diameter, 0.13 NA), and an MM detector fiber optode (400-μm core diameter, 0.50 NA). The size of the speckles could be adjusted by tuning the fiber-SPAD distance via the well-known relationship,^32^ d=λz/D, where d is the average diameter of the speckles, λ is the laser wavelength, z is the fiber-SPAD distance, and D is the MMF detector fiber core diameter. Figure 4(a) shows three images of the speckle pattern recorded by the SPAD camera. As predicted, the average size of the speckles increased when we increased z. We quantified the average diameter of the speckles by calculating the 2D spatial autocorrelation function of each image, as shown in Fig. 4(b). Figure 4(c) shows a linecut from panel (b) at z=106  mm and a Gaussian curve fit that gives the estimated d as the 1/e value of the Gaussian. We repeated the measurement and fitting procedure at increasing z for three different MMF detector fiber core diameters (D=200, 400, and 600  μm). As shown in Fig. 4(d), the measured diameters (solid circles) were in good agreement with the calculated diameters (solid lines) based on the d=λz/D equation. The use of the 2D spatial autocorrelation was limited to speckle diameters larger than the size of a pixel pitch (50×50  μm). We used the calibrated z from the above equation to estimate smaller speckle diameters. We applied this calibration to the milk phantom experiments, for which we used an MM detector fiber with 910-μm core diameter and adjusted z=8.1  mm to obtain d=6.95  μm, matching the diameter of the speckles with the length of the pixel active area.

## 7 Appendix C: Effects of Speckle Size per Pixel on SNR

**Fig. 5:**
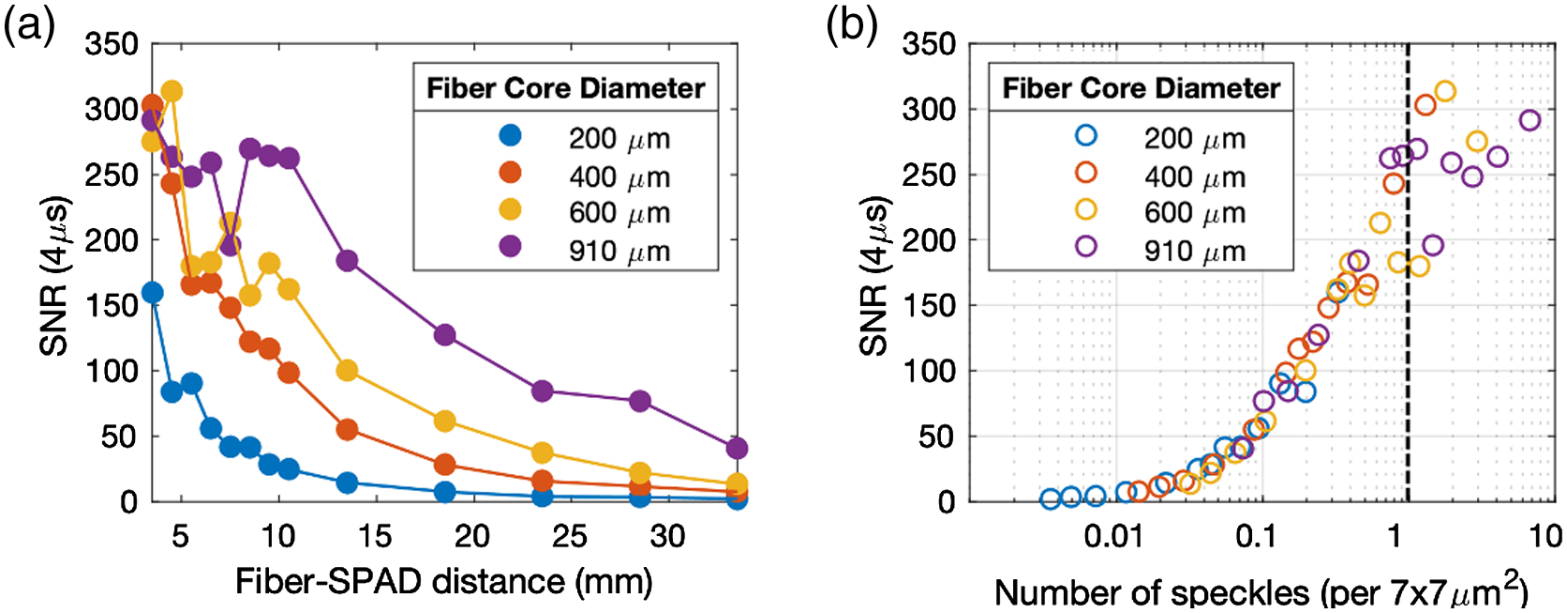
Tuning the diameter of the speckle across the condition of one speckle per pixel. (a) SNR of $g_{2}$ increased with decreasing fiber-SPAD distance. Larger fiber core diameters resulted in higher SNR, particularly prominent for 910-μm fiber core diameter at fiber-SPAD distance of z≤10  mm. (b) SNR of $g_{2}$ versus number of speckles per pixel active area (6.95×6.95  μm). The SNR increased upon reaching the condition of one speckle per pixel active area and achieved a maximum value between S=1 to 10 speckles per pixel active area.

Since the fiber core diameter determines the diameter of the speckles on the SPAD pixel, it is important to investigate how using different fiber core diameters, thus the diameter of the speckles, affects the ensemble SNR of $g_{2}$. We used the liquid phantom setup to perform the SNR versus fiber core diameter study. Figure 5(a) shows that larger fiber core diameters resulted in higher SNR than that of the smaller ones for the same z. The SNR increased with decreasing z for all of the fibers we tested. For the largest core fiber that we tested (which had 910-μm core diameter), the SNR saturated at ∼250 for z<10  mm.

The maximum SNR was achieved when we tuned the diameter of the speckles to be comparable to the length of the pixel active area (6.95×6.95  μm). We examined this by plotting the ensemble SNR of $g_{2}$ (4  μs) (from all fiber core diameters) against the number of speckles per pixel active area, as shown in Fig. 5(b). The number of speckles per pixel active area was calculated as the ratio between the length of the pixel active area and the diameter of the speckles. Figure 5(b) shows that all of the data points fall on a single curve. This suggests that, for a given count rate ($N_{\mathrm{ph}}$) and integration period ($T_{\text{int}}$), the $g_{2}$ SNR in mDCS depends solely on the number of speckles per pixel active area. The SNR gradually increased until it reached one or more speckles per pixel active area [Fig. 5(b)]. Here, S denotes the number of speckles per pixel active area. While β decreased with increasing S, STD also decreased with increasing S due to larger $N_{\mathrm{ph}}$ from having more detected speckles, thus keeping the SNR the same at S≥1  per pixel active area. We observed that β increased if we increased the diameter of the speckles, as predicted, and saturated beyond the Nyquist spatial sampling rate, which we reached when the diameter of the speckles was larger than twice the length of the pixel active area.^35^^,^^36^ This is consistent with the observed onset of saturated SNR for the 910-μm fiber core diameter at z≤10  mm [Fig. 5(a)]. The results presented in this work were achieved using a 910-μm fiber core diameter at z=8.1  mm at which the diameter of the speckles matches the length of the pixel active area (d=a).

## 8 Appendix D: Effects of Hot Pixels in SPAD Camera on SNR

Physical defects in SPAD pixels can effectively increase the DCR due to trapped carriers and after-pulsing. We refer to pixels that have high DCR (>100  Hz) as hot pixels and those with low DCR (<100  Hz) as cool pixels. We identified hot pixels in the kilopixel SPAD camera and investigated their effects on the intensity autocorrelation function $g_{2}$ and SNR of $g_{2}$.

**Fig. 6:**
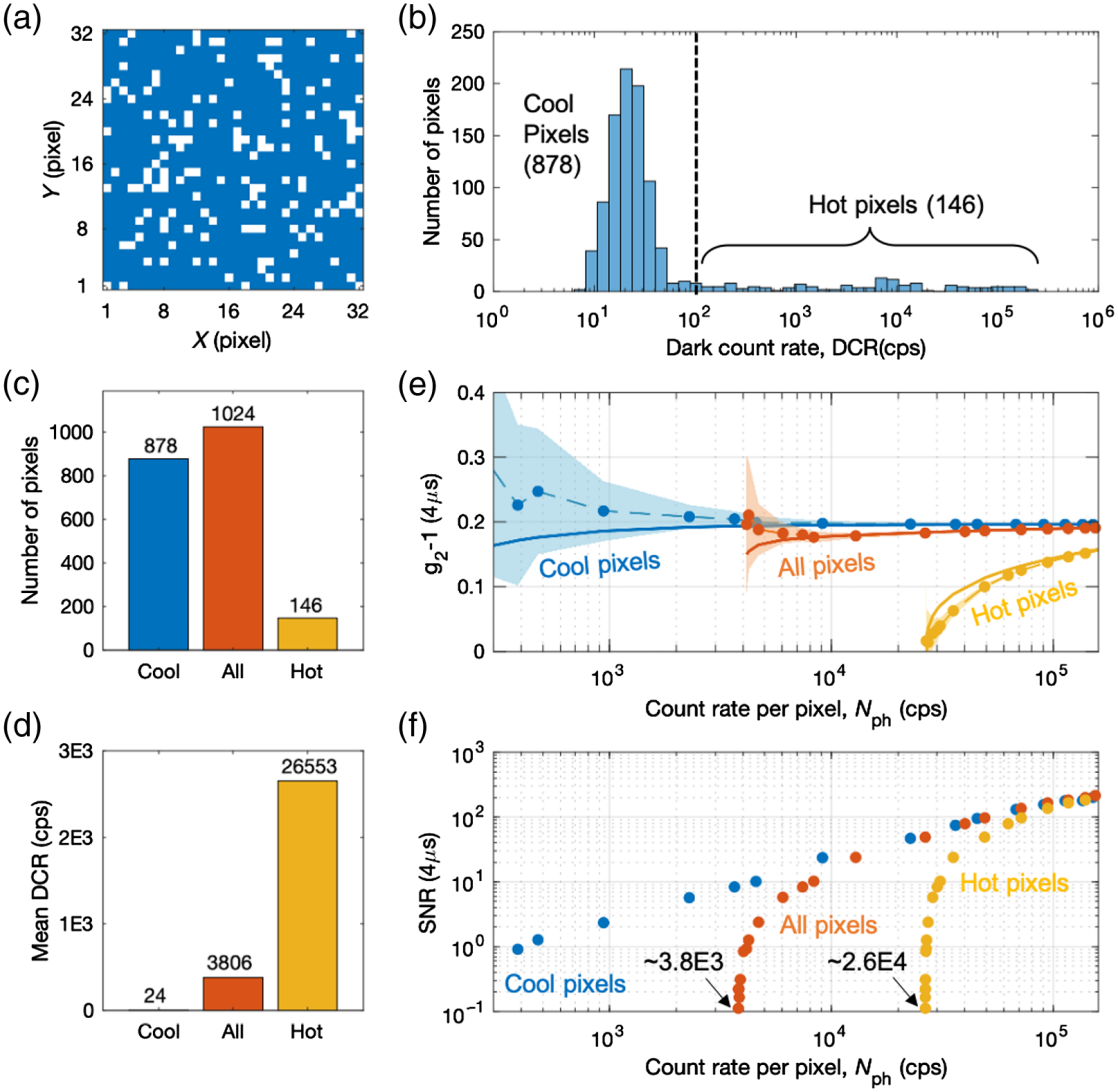
Characterizing hot pixels in SPAD camera. (a) Pixel distribution of 32×32 pixel SPAD camera, where blue corresponds to cool pixels (DCR<100  Hz) and white corresponds to hot pixels (DCR>100  Hz). The SPAD camera aperture was covered with a blank. (b) Distribution of the DCR across all pixels. (c) Distribution of hot and cool pixels. (d) Mean DCR of cool pixels, all pixels, and hot pixels. (e) Measured $g_{2}-1$ (τ=4  μs), or coherence factor β, across selected pixels at varying $N_{\mathrm{ph}}$ ($T_{\text{int}}=50\;\mathrm{ms}$). Here, we used a liquid phantom with source–detector separation of 11 mm and a 785-nm CW laser. The standard deviations are shown by the widths of the color-shaded regions. Solid circles show the measured $g_{2}-1$, and solid lines show the predicted $g_{2}-1$ as calculated using Eq. (6). (f) Measured SNR of $g_{2}$ (τ=4  μs) across selected pixels at varying $N_{\mathrm{ph}}$ (over 160 integration periods).

We characterized the DCR of the SPAD camera by recording the count rate during which the camera’s aperture was covered with a blank. Figures 6(a)–6(c) show the distribution of DCR across 146 hot pixels (white) and 878 cool pixels (blue). The hot pixels make up <15% of the total pixels. Figure 6(d) shows that the mean DCR of hot pixels was 26.6 kcps and that of cool pixels was 24 cps, with a mean DCR of 3.8 kcps across all pixels.

We measured the ensemble average of $g_{2}-1$ (τ=4  μs), which we referred to as the coherence factor β [Fig. 6(e)], and SNR of $g_{2}$ (τ=4  μs) [Fig. 6(f)] from cool pixels (blue), all pixels (orange), and hot pixels (yellow) at decreasing count rate per pixel ($N_{\mathrm{ph}}$). The integration time was $T_{\text{int}}=50\;\mathrm{ms}$, and the ensemble average was performed over 160 integration periods. Figure 6(e) shows that the ensemble average of the cool pixels (blue circles) resulted in β values that remained constant over a wide range of $N_{\mathrm{ph}}$. However, the ensemble average of the hot pixels (yellow circles) resulted in β values that abruptly decreased at decreasing $N_{\mathrm{ph}}$ down to $N_{\mathrm{ph}}∼2.6\times 10^{4}\;\mathrm{cps}$. The ensemble average of all pixels (orange) resulted in β values that were slightly lower than those of the cool pixels down to $N_{\mathrm{ph}}∼3.8\times 10^{3}\;\mathrm{cps}$. The minimum $N_{\mathrm{ph}}$ were different for the three different ensemble averages, which were given by the mean DCR values shown in Fig. 6(d). The corresponding SNR of $g_{2}$ values [Fig. 6(f)] showed a clear decrease in magnitude when the count rates approached the minimum $N_{\mathrm{ph}}$. The SNR versus $N_{\mathrm{ph}}$ plot shows the importance of DCR characterization of the SPAD camera, in particular for DCS measurements at very low $N_{\mathrm{ph}}$.

The decreasing β values with decreasing $N_{\mathrm{ph}}$ were due to DCR contribution from each pixel, which is expressed as

$$\beta \propto \frac{N_{\mathrm{ph}}^{2}}{{\left(N_{\mathrm{ph}}+\mathrm{DCR}\right)}^{2}}.$$ (6)

We calculated the predicted β values for each pixel using Eq. (6); they are plotted in Fig. 6(e) (solid lines) for corresponding ensemble averaging of cool pixels, hot pixels, and all pixels. The calculated β values are consistent with the measurements. In particular, the DCR contribution to the decreasing β values is apparent for the ensemble average of hot pixels.

## 9 Appendix E: Fitting Functions of $g_{2}(\tau )$

The functional form of the intensity autocorrelation function $g_{2}(\tau )$ is determined by the dynamic and optical properties of the scattering media and the geometry surrounding the light source and the detector. In principle, we can predict the functional forms of $g_{2}$ either by directly estimating the resulting phase factor for all of the scattered electric fields for simple systems or by solving the correlation diffusion equation for more elaborate systems. The most commonly used dynamical scattering media in DCS experiments involve scatterers that are undergoing random flow motion (ballistic) or Brownian motion (diffusive). It has been shown^15^^,^^16^^,^^20^^,^^21^ that, in the case of ballistic motion, the functional form can be simplified to $g_{2}(\tau )=1+\beta \;\exp(-\tau^{2}/\tau_{c}^{2})$ at small time lags, $\tau \ll \sqrt{3\mu_{a}/\left(\mu_{s}^{\prime }k_{0}^{2}\alpha v^{2}\right)}$, where $\mu_{a}$ and ${\mu_{s}}^{\prime }$ are the absorption and reduced scattering coefficients of the medium, $k_{0}$ is the wavenumber of the light, α is the percentage of light scattering events from moving scatterers, and v is the standard deviation of the speed of the scatterers. Similarly, in the case of diffusive motion, $g_{2}(\tau )=1+\beta \;\exp(-\tau /\tau_{c})$ at small time lags, $\tau \ll \mu_{a}/\left(2\mu_{s}^{\prime }k_{0}^{2}\alpha D_{b}\right)$, where $D_{b}$ is the diffusion coefficient of the moving scatterers.

**Fig. 7:**
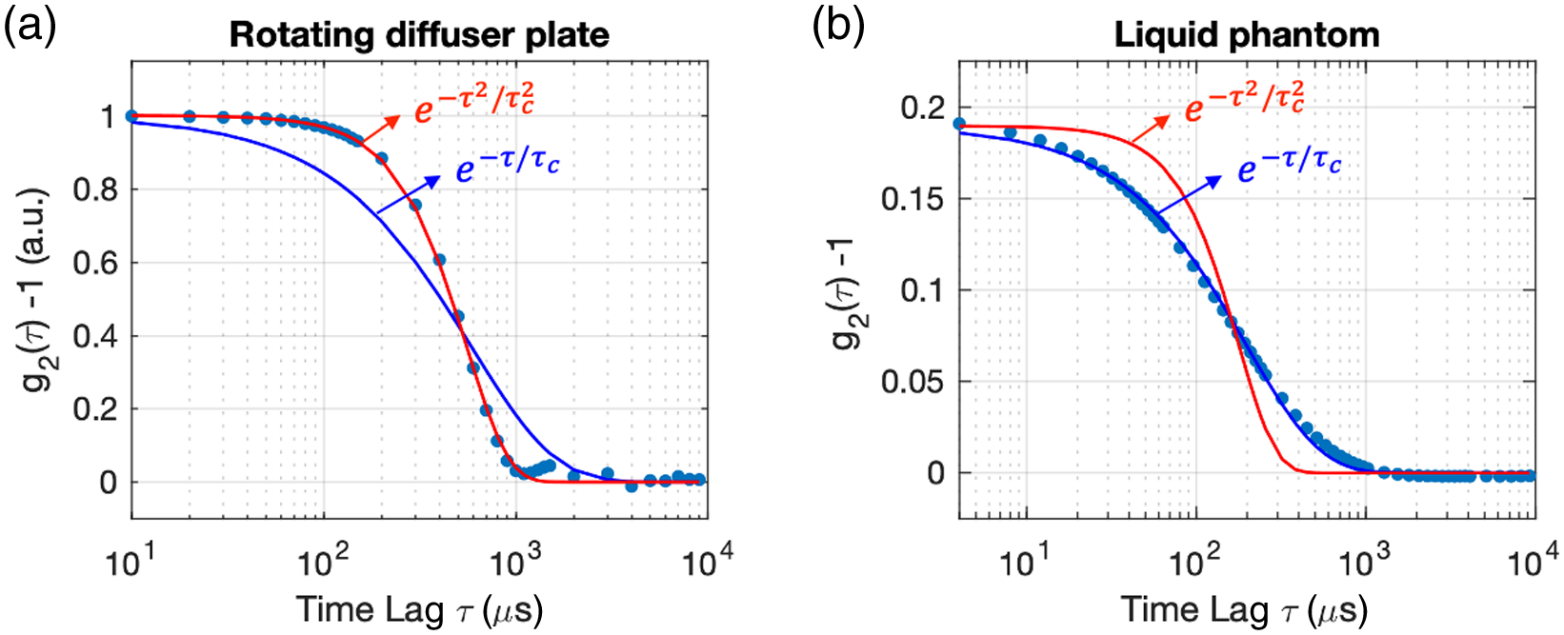
Fitting functions of $g_{2}(\tau )$. (a) Normalized $g_{2}(\tau )-1$ (solid circles) measured from the rotating diffuser plate and two different functional forms of $g_{2}(\tau )$ that were used to fit the measured data: $g_{2}(\tau )-1=\beta \;\exp(-\tau^{2}/\tau_{c}^{2})$ (red curve) and $g_{2}(\tau )-1=\beta \;\exp(-\tau /\tau_{c})$ (blue curve). The $g_{2}$ curve was normalized by the value of $g_{2}$ at τ=10  μs. The measured $g_{2}(\tau )$ can be fitted well with $g_{2}(\tau )-1=\beta \;\exp(-\tau^{2}/\tau_{c}^{2})$, where $\tau_{c}=557\;\mu s$, consistent with the ballistic motion dynamics of the rotating diffuser plate. We measured β=0.7 with the rotating diffuser plate setup. The resulting β was higher than the value expected from the case of unpolarized light (β=0.5) likely due to a polarization bias in the detected light. The speckle diameter was tuned to be three times larger than the pixel size (d>3a). (b) $g_{2}(\tau )-1$ (solid circles) measured from the liquid phantom and two functional forms of $g_{2}(\tau )$ (solid curves). The measured $g_{2}(\tau )$ can be fitted well with $g_{2}(\tau )-1=\beta \;\exp(-\tau /\tau_{c})$, where $\tau_{c}=197\;\mu s$, consistent with the diffusive motion dynamics of the liquid phantom. We measured β=0.2 with the liquid phantom setup. The resulting β was lower than the value expected from the case of unpolarized light (β=0.5) and results from having the speckle diameter matched with the pixel active area (d=a). The maximum value for unpolarized light (β=0.5) can be obtained by increasing the speckle diameter beyond the Nyquist rate (d>2a).

In our work, the scattering media were a rotating diffusive plate [Fig. 1(a)] and a liquid phantom [Fig. 1(e)], and the details of the experimental setups were discussed in Appendix A. Figure 7 shows the normalized $g_{2}(\tau )$ (solid circles) measured from the rotating diffuser plate [Fig. 7(a)] and the liquid phantom [Fig. 7(b)]. We also plotted the functional forms of $g_{2}(\tau )$ that were used to fit the measured data: $g_{2}(\tau )-1=\beta \;\exp(-\tau^{2}/\tau_{c}^{2})$ (red curve) and $g_{2}(\tau )-1=\beta \;\exp(-\tau /\tau_{c})$ (blue curve). As shown in Fig. 7, the intensity autocorrelation function of the rotating diffuser plate can be fitted well with the ballistic motion functional form $\exp(-\tau^{2}/\tau_{c}^{2})$ and that of the liquid phantom with the diffusive motion functional form $\exp(-\tau /\tau_{c})$ over a wide range of time lags. The fitting results are consistent with the fact that the scatterers in the rotating diffusive plate behave like ballistic scatterers and those in the liquid phantom behave like diffusive scatterers.

## Code, Data, and Materials Availability

The data that support the findings of this study are available from the corresponding author on reasonable request.
